# The Expression of Neuroendocrine Markers in a Small Subset of Ameloblastoma with Implications of Clusterin

**DOI:** 10.3390/cells14030224

**Published:** 2025-02-05

**Authors:** Hiromasa Hasegawa, Takanaga Ochiai, Rita R. Roy, Katsumitsu Shimada

**Affiliations:** 1 Hard Tissue Pathology Unit, Graduate School of Oral Medicine, Matsumoto Dental University, Shiojiri 399-0781, Japan; 2 Department of Oral Pathology/Forensic Odontology, School of Dentistry, Aichi Gakuin University, Nagoya 464-8651, Japan; 3 Department of Oral Pathology, Division of Oral Pathogenesis & Disease Control, School of Dentistry, Asahi University, Mizuho 501-0296, Japan; t-ochiai@dent.asahi-u.ac.jp; 4 Department of Physiology, Matsumoto Dental University, Shiojiri 399-078, Japan; rani.roy.rita@mdu.ac.jp; 5 Department of Clinical Pathophysiology, Matsumoto Dental University, Shiojiri 399-078, Japan; katsumitsu.shimada@mdu.ac.jp

**Keywords:** ameloblastoma, neuroendocrine differentiation, synaptophysin, insulinoma-associated protein 1, clusterin

## Abstract

Immunohistochemically, ameloblastomas often express CD56; however, novel neuroendocrine markers such as synaptophysin (SYP), insulinoma-associated protein 1 (INSM1), and chromogranin A (CgA) remain unexplored. We analyzed 36 ameloblastoma specimens for CD56, SYP, CgA, and clusterin (CLU) and examined limited samples for INSM1 expression by performing immunohistochemistry, transmission electron microscopy, and reverse transcriptase-polymerase chain reaction. Our findings indicate that the limited cells were positive for CD56, SYP, CgA, INSM1, and CLU expression in 72% (26/36), 14% (5/36), 0% (0/40), 80% (4/5), and 22% (8/36) of the cases, respectively. CD56 expression correlated with older age, but not with subtype, SYP, and CLU expression. However, SYP-positive cases were exclusively found in CD56- and CLU-positive cases, and SYP and CLU expression were significantly correlated. Selected cases had dense-core granules and *NCAM1* and *SYP* mRNA expression. This study is the first to suggest neuroendocrine differentiation in ameloblastomas, as indicated by SYP and INSM1 immunoexpression and the presence of dense-core granules, which are consistent with the recent World Health Organization classification of Head and Neck Tumors guidelines. SYP-positive and CgA-negative phenotypes may characterize neuroendocrine differentiation in ameloblastoma. Although the underlying molecular mechanism remains unclear, CLU expression may be associated with neuroendocrine differentiation.

## 1. Introduction

Ameloblastomas are the most prevalent and benign but are locally aggressive epithelial tumors that affect the jawbones. These tumors comprise columnar epithelial and stellate epithelial cells that resemble ameloblasts and stellate reticulum cells of the enamel organs, respectively [1].

Cluster of differentiation 56 (CD56), also known as neural cell adhesion molecule (NCAM) and initially identified as antigen Leu-19, is expressed across various normal cell types and tumor entities, including natural killer (NK) cells, neural cells, myogenic progenitor cells, NK cell lymphomas, and neuroendocrine carcinomas [2,3,4]. Additionally, CD56 expression has been observed in non-neuroendocrine thyroid neoplasms, including follicular adenoma and papillary thyroid carcinoma [5].

In odontogenic lesions, CD56 expression has been observed in ameloblastomas, with an incidence of 74–97% and an average expression rate of approximately 87% [6,7,8]. In contrast, odontogenic keratocysts, which are classified as odontogenic cysts in the recent WHO classification [1], show less consistent CD56 expression, with reported frequencies of 5–50% and an overall average of approximately 31%. These findings suggest that CD56 is upregulated in certain neoplastic contexts [6,7,8]. However, information regarding the biological significance and neuroendocrine properties of CD56-expressing ameloblastomas remains limited.

One study by Kusafuka et al. [6] documented the immunoexpression of neurogenic differentiation 1 (NEUROD1), N-cadherin, and CD56 in ameloblastomas. In small cell lung carcinoma, transcription factors such as NEUROD1, achaete-scute family bHLH transcription factor 1 (ASCL1), POU class 2 homeobox 3 (POU2F3), and Yes1-associated transcriptional regulator (YAP1) are used for sub-classification [9]. NEUROD1 is expressed in approximately 50% of small cell lung carcinoma cases; however, this expression is not universal. In contrast, POU2F3 is detected in less than 10% of cases and is typically restricted to ASCL1/NEUROD1 double-negative tumors [10]. These observations imply that NEUROD1 alone is insufficient as a neuroendocrine marker. To date, there are no definitive reports showing that ameloblastoma expresses endocrine markers, and it remains unclear whether these tumor cells can differentiate into endocrine cells.

In the previous World Health Organization (WHO) classification of Head and Neck Tumors (4th edition), synaptophysin (SYP), chromogranin A (CgA), and CD56 have been recommended as immunohistochemical markers of neuroendocrine differentiation [11]. However, the updated WHO classification discourages the use of CD56 due to its non-specificity for neuroendocrine neoplasms (NENs) and instead advocates for CgA, SYP, and insulinoma-associated protein 1 (INSM1) as novel markers of neuroendocrine differentiation in the head and neck region [12]. 

Additionally, clusterin (CLU), a ubiquitous multifunctional secretory sulfated glycoprotein, plays roles in apoptosis and cell differentiation and is overexpressed in various tumors, including neuroendocrine tumors [13]. CLU is associated with neuroendocrine differentiation [14] and ameloblastic differentiation during odontogenesis [15,16]. 

This study aims to clarify whether CD56 positivity in ameloblastomas is indicative of neuroendocrine differentiation or if it represents a distinct phenomenon unrelated to the neuroendocrine lineage. By addressing this question, we hope to contribute to a more nuanced understanding of CD56 expression in the context of ameloblastoma biology. Therefore, first of all, we investigated the nature of ameloblastomas using the classic and revised neuroendocrine markers, CD56, SYP, CgA, and INSM1, as well as the correlation between CLU and the expression of neuroendocrine markers.

## 2. Materials and Methods

### 2.1. Sample Selection 

Formalin-fixed, paraffin-embedded (FFPE) human samples were obtained from the archives of the Department of Surgical Pathology at Matsumoto Dental University Hospital. All the patients were diagnosed with ameloblastoma at the Department of Surgical Pathology. After examining hematoxylin and eosin (HE)-stained sections, the most representative cases were selected according to the criteria established in the World Health Organization classification of Head and Neck Tumors (5th edition) [1]. Decalcified specimens (treated with a rapid decalcification solution) were excluded from the selected samples to ensure their suitability for immunohistochemical and molecular analyses. Finally, 36 patients were included in the study (Supplemental Table S1). Because these cases were small biopsies or portions of surgical specimens, only a limited number of specimens were available for analysis, particularly for ultrastructural and molecular analyses.

This study was conducted in compliance with the principles of the 2008 Declaration of Helsinki, and the protocol was approved by the Ethics Committee of Matsumoto Dental University (approval number 0166). Informed consent was obtained through an opt-out method as all samples were archived FFPE specimens.

### 2.2. Immunohistochemical Analyses 

Three-micrometer-thick histological sections were prepared from the FFPE samples. Table 1 lists the antibodies used to confirm neuroendocrine differentiation. Heat retrieval methods using a pressure cooker with a heat-induced epitope retrieval solution at either pH 6.0 (#415281, Nichirei Bioscience Inc., Tokyo, Japan) or 9.0 (#41520, Nichirei Bioscience Inc., Tokyo, Japan) were employed. Following the retrieval treatment, endogenous peroxidase activity was blocked by incubating the sections in 3% aqueous H_2_O_2_ for 10 min, followed by a 30 min incubation in a protein block solution (Agilent Technologies, Santa Clara, CA, USA) to prevent nonspecific reactions. Sections were incubated with primary antibodies for an hour at 22 °C, followed by incubation with a horseradish peroxidase-conjugated secondary antibody (#414152F, Nichirei MAX-PO Multi [host: goat], Nichirei Bioscience Inc.) for 30 min at 22 °C. The samples were first stained with 3,3′-diaminobenzidine tetrahydrochloride (Agilent Technologies, Santa Clara, CA, USA) and then counterstained with hematoxylin. Pancreatic islet cells served as external positive controls for SYP, CgA, and INSM1. Normal lymph node and brain tissue served as positive controls for CD56 and CLU, respectively. Negative control sections were prepared according to the aforementioned procedure, excluding primary antibodies, to identify non-specific reactions.

Finally, thirty-six cases were examined for CD56, SYP, CgA, and CLU, and five SYP-positive cases were further examined for INSM1. We attempted to determine whether the estimated ratio of each neuroendocrine marker exceeded 30% based on the definition of mixed neuroendocrine–non-neuroendocrine neoplasm (MiNEN) [13].

### 2.3. Transmission Electron Microscopic Analysis

FFPE specimens from two SYP-positive cases (cases 4 and 25) were used for transmission electron microscope (TEM) analysis. Based on the examination of the HE-stained sections, target areas containing SYP-positive cells were manually dissected, and small cubes were prepared for TEM analysis. After deparaffinization and washing with a 0.1 M cacodylate buffer, the specimens were fixed in Karnovsky’s solution (2% paraformaldehyde and 2.5% glutaraldehyde in a 0.1 M sodium phosphate buffer), post-fixed in 1% osmium acid, dehydrated, and embedded in Epon 812 resin (Nisshin EM Co., Ltd., Tokyo, Japan). Ultrathin sections mounted on uncoated copper grids were stained with uranyl acetate and lead citrate. The sections were examined using a transmission electron microscope (H-7600, Hitachi High-Tech Corporation, Tokyo, Japan).

### 2.4. RNA Isolation and Reverse Transcriptase-Polymerase Chain Reaction

To assess the mRNA expression of the *NCAM1*, *SYP*, and *CHGA* genes, we selected cases 4 (CD56^+^/SYP^+^/CgA^−^), 14 (CD56^+^/SYP^−^/CgA^−^), and 23 (CD56^−^/SYP^−^/CgA^−^). Total RNA was isolated from FFPE samples using the RNeasy Mini Kit (#74904; QIAGEN N.V., Hilden, Germany) following the manufacturer’s protocol. Complementary DNA (cDNA) synthesis was performed using 0.025 µg random primers (#48190011; Invitrogen/Thermo Fisher Scientific, Waltham, MA, USA) and the SuperScript III reverse transcriptase (#18080-093, Invitrogen/Thermo Fisher Scientific, Waltham, MA, USA) in a final volume of 20 µL.

For polymerase chain reaction (PCR) amplification, 50 ng of cDNA was diluted to a final volume of 50 µL, containing 25 µL of Premix Ex Taq Version 2.0 (Takara Bio Inc., #RR003A, Kusatsu, Shiga, Japan) and 4 µL of 4 µM primer mix. The PCR program comprised initial denaturation at 95 °C for 2 min, followed by 30 cycles of denaturation at 95 °C for 30 s, annealing at 55 °C for 30 s, and extension at 74 °C for 30 s, with a final elongation at 74 °C for 7 min. Table 2 lists the primer sequences used for PCR. To normalize the mRNA expression levels, we used the human glyceraldehyde-3-phosphate dehydrogenase (*GAPDH*) primer from the Human Housekeeping Gene Primer Set (#3790; Takara Bio Inc., Kusatsu, Shiga, Japan). We selected *GAPDH* as an internal control due to its consistent and stable expression in human tissues. The *GAPDH* primers were used under the same RT-PCR conditions as those described for the target genes to ensure the accurate and reliable quantification of mRNA expression. The PCR products were then resolved on a 2% agarose gel.

### 2.5. Statistical Analysis

After assessing the distribution of patients’ ages using the Shapiro–Wilk normality test, correlations between age and positivity of neuroendocrine markers were analyzed using the Mann–Whitney U test. Fisher’s exact test was used to evaluate the correlations between subtypes and the immunoreactivity of CD56, SYP, and CLU; immunoreactivity between CD56 and SYP; between sex and the immunoreactivity of CD56 and SYP; and between the immunoreactivity of CLU and those of CD56 and SYP. All statistical analyses were performed using EZR (Saitama Medical Center, Jichi Medical University, Saitama, Japan) [14], a graphical user interface for R (www.r-project.org, accessed on 1 March 2019) (R Foundation for Statistical Computing, Vienna, Austria). Statistical significance was set at *p* < 0.05.

## 3. Results

### 3.1. Clinical and Histological Summaries 

The mean age of the patients was 44.2 years (range, 10–81 years). The male/female ratio was 1.6:1 (Supplementary Table S1).

**Figure 1:**
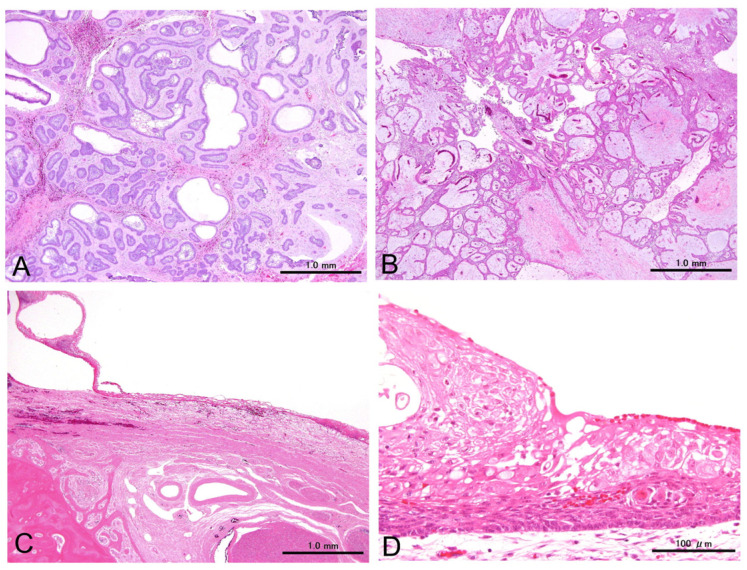
Representative cases of conventional ameloblastomas displaying follicular (**A**) and plexiform (**B**) patterns, as well as an unicystic ameloblastoma showing a single cyst (**C**,**D**). All cases comprise columnar and stellate cells that resemble enamel organs. Scale bars: 1.0 mm (**A**–**C**) and 100 µm (**D**).

The 36 patients exhibited conventional or unicystic subtypes, accounting for 21 (58%) and 15 (42%) cases, respectively. The conventional ameloblastomas were composed of variably mixed follicular and plexiform patterns. Follicular dominant and plexiform dominant cases were twelve (57%) and nine (43%), respectively. The tumor cells were characterized as peripheral cuboidal and stellate reticulum-like cells, along with variable cystic changes, squamous metaplasia, and keratinization (Figure 1). 

### 3.2. Immunohistochemical Results

**Figure 2:**
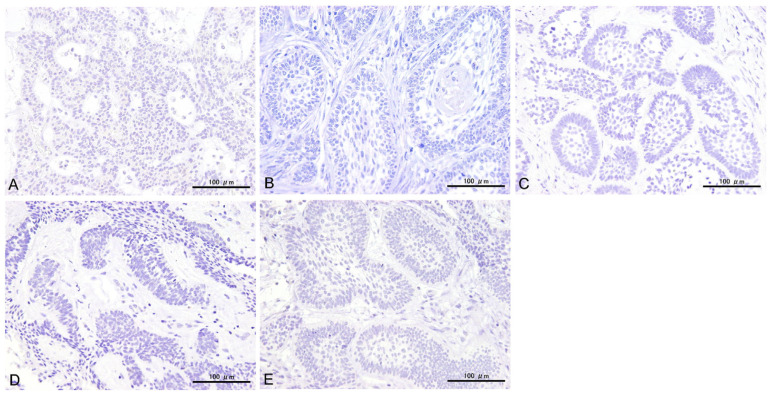
Representative images of negative control slides for CD56 (**A**), SYP (**B**), CgA (**C**), INSM1 (**D**), and CLU (**E**). Scale bars: 100 μm.

All the antibodies investigated in this study showed varying positive rates. CD56, SYP, CgA, INSM1, and CLU were detected in 72% (26/36), 14% (5/36), 0% (0/40), 80% (4/5), and 22% (8/36) of specimens, respectively. All negative control slides showed no reactivity in any of the areas (Figure 2).

**Figure 3:**
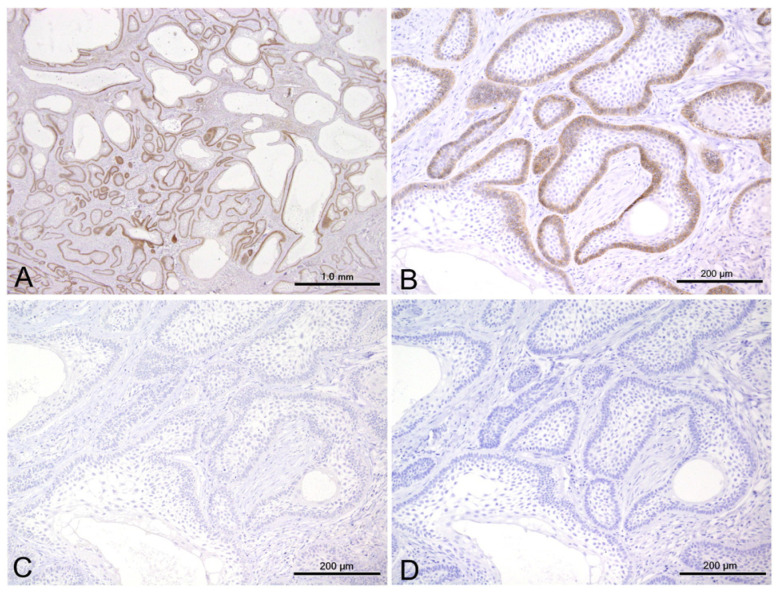
Representative examples showing CD56 positivity (**A**,**B**) and SYP (**C**) and CgA (**D**) negativity. Scale bars: 1.0 mm (**A**) and 200 μm (**B**–**D**).

Positive cytoplasmic reactions to CD56 were mostly found in peripherally located columnar cells mimicking ameloblasts and in some stellate reticulum-like cells. The CD56-positive areas ranged from focal to diffuse, depending on the case. Even in cases with extensive positive reactions, the positive cells did not constitute more than 30% of the entire tumor cell population. However, most CD56-positive specimens (81%, 21/26) were negative for SYP and CgA (Figure 3). 

**Figure 4:**
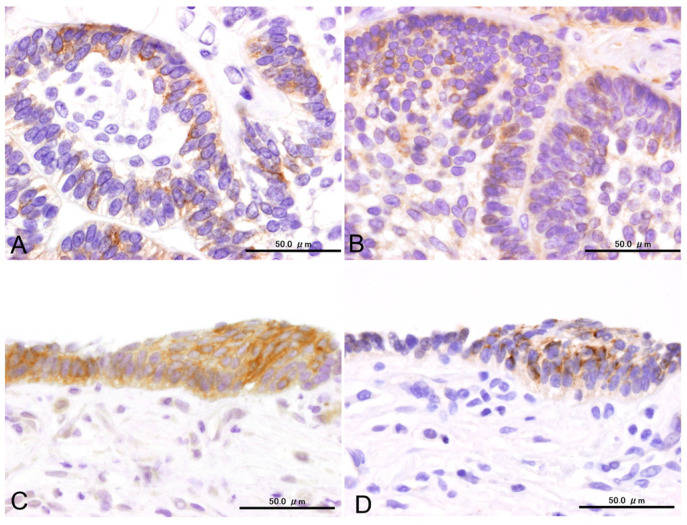
Immunoexpression of SYP and CLU in conventional (**A**,**B**) and unicystic (**C**,**D**) subtypes. Scale bars: 50.0 μm.

Cytoplasmic SYP reactions were observed in the peripheral cells, in some stellate cells within the nests of the conventional subtype, and in the cells lining the cystic spaces of the unicystic subtype. The ratio of SYP-positive cells could not be accurately calculated because of the limited specimens. Nevertheless, SYP-positive cells were scattered in limited tumor nests and appeared to comprise no more than 30% of the entire tumor (Figure 4).

We also observed cytoplasmic CLU reactions in the columnar and stellate cells of most SYP-positive specimens. The distribution pattern of CLU was similar to that of SYP, with positive reactions particularly evident throughout the layers of the unicystic subtypes (Figure 4).

**Figure 5:**
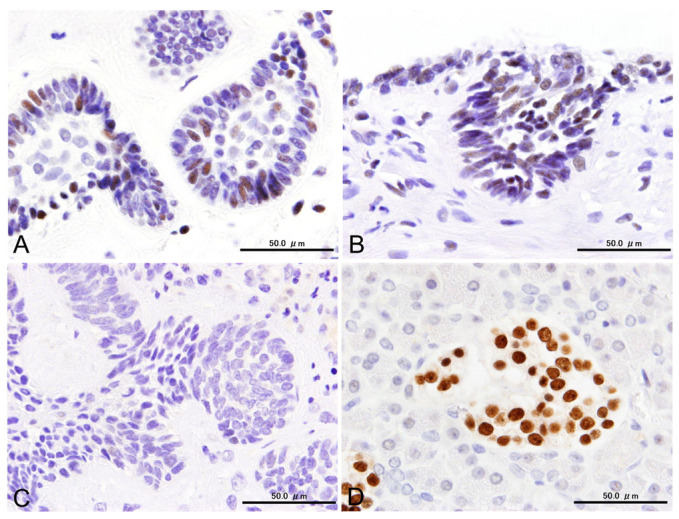
Nuclear INSM1 positivity in conventional (**A**) and unicystic (**B**) subtypes. An example of an INSM1-negative case with SYP-positive is shown (**C**), alongside strong INSM1 positivity in pancreatic islet cells (**D**). Scale bars: 50.0 μm.

INSM1-positive reactions could be found in SYP-positive specimens except for one case; however, the reactivity of ameloblastomas was weaker than that of pancreatic islet cells. Weakly positive INSM1 staining was observed in the nuclei of columnar cells located in the periphery and in some stellate cells in SYP-positive cases (Figure 5).

Table 3 summarizes the cases with the co-expression of CD56 and SYP, including five ameloblastomas. In contrast to SYP positivity, CgA positivity was consistently negative in all the cases. INSM1 was expressed in most instances and was positive in four out of five ameloblastomas. CLU expression was observed in all SYP-positive cases. Notably, five cases with consistent positivity for CD56, SYP, and CLU were of the conventional and unicystic subtypes.

### 3.3. Statistical Results for Immunohistochemistry

**Figure 6:**
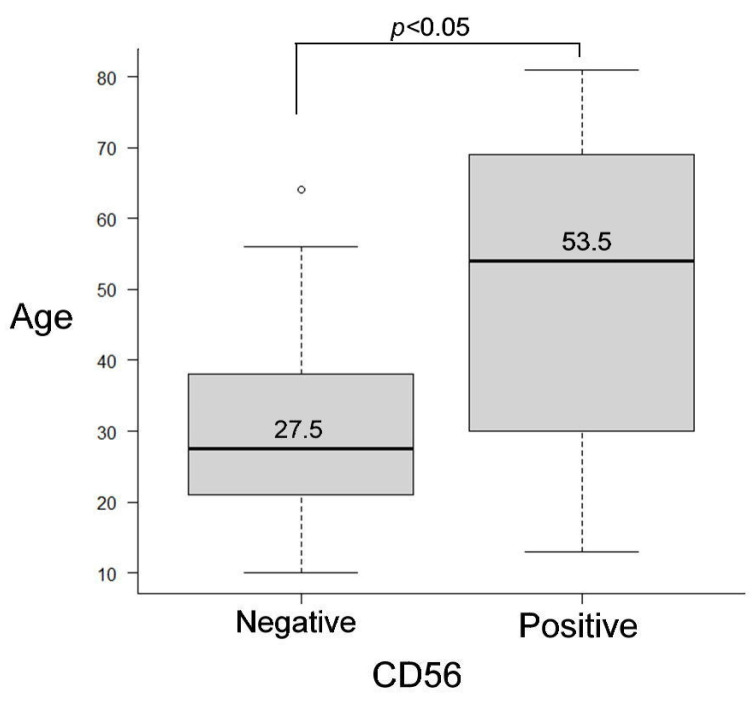
Box plot bars showing statistically significant differences between the CD56-negative and CD56-positive groups (*p* < 0.05).

The Mann–Whitney U test revealed that the patients in the CD56-negative group had a median age of 27.5 years (25th percentile: 21.3 years, 75th percentile: 37.8 years), ranging from 10 to 64 years. In contrast, patients in the CD56-positive group had a median age of 53.5 years (25th percentile, 28.5 years; 75th percentile, 69.5 years), ranging from 13 to 81 years. The difference between the two groups was statistically significant (*p* < 0.05), suggesting that CD56 expression was associated with older patient age (Figure 6). However, we observed no statistically significant difference between the SYP-negative and SYP-positive groups (*p* = 0.156).

The expression of CD56 and SYP was not significantly associated with sex. Regarding the association of CD56, SYP, and CLU expression with subtypes, no statistical significance was noted (Table 4).

We observed no significant relationship between CD56 and SYP expression (*p* = 0.268). As shown in Table 5, CD56 expression was associated with CLU expression; however, this association was not statistically significant (*p* = 0.0756). SYP expression significantly correlated with CLU, as five out of eight CLU-positive cases were SYP-positive compared to zero out of twenty-eight CLU-negative cases (*p* < 0.001).

### 3.4. Transmission Electron Microscopic (TEM) Findings

**Figure 7:**
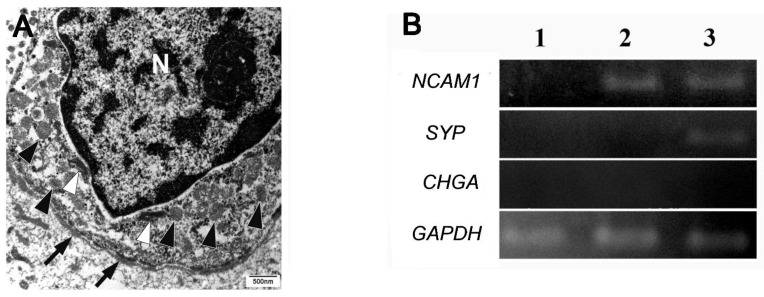
Transmission electron microscopy findings. Peripherally located columnar cells contain few tonofilaments (white arrowheads) and some dense-core granules (arrowheads) measuring approximately 200 nm in diameter. N indicates the nucleus, and arrows denote the basement membrane. Scale bar: 500 nm (**A**). Agarose gel electrophoresis showing positive bands for *NCAM1* and *SYP* in lane 3 (case 4: CD56^+^/SYP^+^/CgA^−^), *NCAM1* in lane 2 (case 14: CD56^+^/SYP^−^/CgA^−^), and no bands in lane 1 (case 23: CD56^−^/SYP^−^/CgA^−^) (**B**).

Transmission electron microscopy revealed that the columnar cells located at the periphery contained dense-core granules along with a few intermediate tonofilaments. The granules were homogeneously dense, round, or slightly irregular in shape (Figure 7A). Morphometric analysis of the dense-core granules revealed a mean diameter of 200.5 nm, ranging from 112.2 nm to 287.1 nm.

### 3.5. mRNA Expressions

NCAM1 and SYP mRNAs were detected in specimens positive for CD56 and SYP, respectively. However, CHGA mRNA was not detected in any of the three tested specimens (Figure 7B).

## 4. Discussion

We examined neuroendocrine markers, including CD56, SYP, and CgA, in 36 ameloblastomas and identified five specimens that exhibited the immunoexpression of CD56 and SYP, suggesting neuroendocrine differentiation in ameloblastomas. Additionally, the immunoexpression of INSM1, a second-generation neuroendocrine marker, in SYP-positive ameloblastomas further supports this finding, along with the ultrastructural presence of dense-core granules. To the best of our knowledge, this is the first study to suggest that a small subset of ameloblastomas may exhibit neuroendocrine characteristics.

Most immunohistochemical markers have limitations, and established proteins indicative of neuroendocrine differentiation are no exception. CgA, SYP, and CD56 expressions are routinely checked in surgical diagnostics, and positive immunoreactivity for these markers is commonly observed in neuroendocrine neoplasms. However, in some cases, non-neuroendocrine neoplasms can be misdiagnosed as neuroendocrine neoplasms due to non-specific marker expression [15]. In some carcinomas (such as cutaneous basal cell carcinoma), CD56 immunopositivity may serve as a prognostic marker in patients with a high risk of recurrence [16]. However, the biological significance of CD56 expression in ameloblastoma remains unclear. 

All CD56-positive cases were conventional and unicystic ameloblastomas, which are classified as benign odontogenic tumors [1]. We found no statistical difference in CD56 expression between these two subtypes. It is well known that CD56 is expressed in many non-epithelial and epithelial non-odontogenic malignant tumors [2,3,4,5]. Similarly, CD56 appears to be expressed in ameloblastic carcinoma [17]. However, the biological impact of CD56 seems to be limited, as more than 70% of ameloblastomas in our series were positive, and odontogenic cysts can also show CD56 positivity [6,7,8].

In this study, patients with CD56-positive ameloblastomas were significantly older than those with CD56-negative tumors. Although we cannot explain this result based on the results of the current study, CD56 expression may be linked to age-associated molecular or genetic changes in ameloblastomas. Aging causes various events such as genomic instability, telomere attrition, and epigenetic alterations, all of which can affect transcriptional activity [18,19]. Although further investigation is needed, specific signaling pathways or genetic mutations, which are more prevalent in older individuals, may possibly contribute to CD56 expression.

In ameloblastomas, the cytoplasm of peripherally located tumor cells comprises scattered vesicles, bundles of tonofilaments, and microtubules. Notably, numerous dense-core secretory granules have been observed, primarily situated in the basal regions of these cells, along with glycogen and lipid droplets [20]. However, studies have not specifically described neuroendocrine granules in ameloblastomas.

Detecting target structures in a limited area of a lesion using TEM is challenging. However, the ability to target specific areas for TEM analysis facilitates the retrospective ultrastructural examination of rare lesions preserved in paraffin blocks. Despite the generally poor preservation of fine morphology in the TEM analyses of FFPE samples, neuroendocrine granules remain sufficiently well preserved to allow for meaningful observation [21]. Capella et al. [22] reported that in small cell carcinoma of the breast, neuroendocrine granules vary in size, with mean diameters ranging from 80 to 400 nm, depending on the granule type. The dense-core granules observed in the peripheral cells of ameloblastomas were approximately 200 nm in size (ranging from 111 to 287 nm) and aligned with type II neuroendocrine granules, which were homogeneously dense, medium-sized (120 to 250 nm), and round or slightly irregular in shape.

These secretory granules may also contain enamel-related proteins. Selvam et al. [23] demonstrated that the peripheral tumor cells of some, but not all, ameloblastomas expressed ameloblastin, which highlighted the heterogeneity in protein expression within these tumors. Conversely, Crivelini et al. [24] reported that all four ameloblastoma cases investigated were consistently negative for enamel matrix proteins, including amelogenin, amelotin, ameloblastin, and odontogenic ameloblast-associated protein. However, other mixed odontogenic tumors demonstrated varying positivities for these proteins. Further supporting these findings, Tsujigiwa et al. [25] identified the expression of amelogenin mRNA in ameloblastoma specimens; however, despite its transcriptional presence, no amelogenin protein was detected, as confirmed by in situ hybridization and RT-PCR. This discrepancy between mRNA and protein expressions, as well as the limited or inconsistent expression of enamel-related proteins (particularly ameloblastin), suggests that ameloblastomas may exhibit a diverse range of differentiation. Collectively, these observations underscore the biological heterogeneity of ameloblastomas and indicate that the dense-core granules may be something other than enamel proteins.

In the 2022 World Health Organization classification of endocrine and neuroendocrine tumors, the essential diagnostic criteria for neuroendocrine neoplasms in non-endocrine organs include diffuse and intense expression of cytokeratins and either CgA or two other neuroendocrine markers, such as SYP and INSM1 [26]. Similarly, the latest edition of WHO classification of Head and Neck Tumors considers CD56 a non-specific biomarker and therefore discourages its use for identifying neuroendocrine neoplasms. Instead, the guidelines recommend CgA, SYP, and INSM1 expressions as immunohistochemical markers for neuroendocrine neoplasms of the head and neck region [12].

INSM1, a member of the second-generation neuroendocrine marker family, has expanded diagnostic toolkits. The combination of INSM1, SYP, and CgA is readily available for routine histopathological assessments [15]. INSM1 is a highly sensitive and specific single marker for neuroendocrine differentiation across all tumor types in the head and neck region [27]. The sensitivity of INSM1 is comparable to that of SYP and CD56 but markedly surpasses that of CgA in many cases. In large-cell neuroendocrine carcinoma, CD56 and SYP demonstrated greater sensitivity than INSM1, whereas CgA showed lower sensitivity. However, even carcinoid tumors expressing SYP and CgA may occasionally lack INSM1 expression [28].

Most immunohistochemical markers have limitations, and established proteins indicative of neuroendocrine differentiation are of no exception. Despite its utility, INSM1 is ectopically expressed in a limited number of squamous cell carcinomas, acinic cell carcinomas, mucoepidermoid carcinomas, and sinonasal adenocarcinomas of the head and neck [27]. SYP also shows ectopic or aberrant expression in adenocarcinomas of various origins, including malignant melanomas or sarcomas [15]. Interestingly, the ectopic expression of neuroendocrine markers is usually observed in malignant neoplasms but not in benign epithelial neoplasms. The ectopic expression of SYP and INSM1 in ameloblastomas seems to be a unique example of SYP and INSM1 expression. These unusual immunoreactions may be diagnostic pitfalls for small biopsy specimens. 

Mixed neuroendocrine/non-neuroendocrine neoplasms (MiNENs) are neoplasms in which either component constitutes at least 30% of the lesion. Consequently, cases in which one of the two components represents only a minority of the tumor burden should not be classified as mixed neoplasms [13]. Based on the MiNEN criteria, we assessed whether the volume of the SYP-positive area exceeded 30%. Consequently, all SYP-positive cells accounted for less than 30% of the cutoff value. Given the recent understanding of neuroendocrine characteristics, the immunoexpression of SYP and INSM1 in a limited number of tumor cells in this study signifies genuine neuroendocrine differentiation in ameloblastomas. The presence of dense-core granules further supports the immunohistochemical results. However, the underlying molecular mechanisms of this unique phenomenon remain unknown.

CLU, encoded by a 16-kilobase gene located on chromosome 8p21-p12, is a potent extracellular chaperone that prevents protein aggregation and precipitation caused by physical or chemical stressors [29,30]. It has various biological functions, including roles in cellular survival, cell cycle arrest, and the modulation of apoptosis [31]. Initially, CLU was considered associated with neuroendocrine differentiation because its expression was limited to neuroendocrine cells in the normal colorectal epithelium [32]. Although well-differentiated neuroendocrine tumors strongly express CLU at many anatomical sites, other tumor types, such as anaplastic large-cell lymphoma, follicular dendritic cell sarcoma, and tenosynovial giant cell tumors, have also expressed CLU [33]. 

CLU is hypothesized to regulate cell proliferation and differentiation in various tissues, including the development and differentiation of neural stem cells [34]. *CLU* expression is elevated during the secretory stage of odontogenesis, where it is co-expressed with proteins such as ameloblastin and enamelin [35]. Interestingly, the *CLU* mRNA levels significantly increased before the upregulation of ameloblastin and amelogenin [36]. Immunolabeling studies indicated that CLU expression is not restricted to secretory ameloblasts but is also observed in postnatal mouse tissues, including the dental laminae and basal cells of the oral epithelium; this suggests that CLU plays a role in epithelial proliferation and differentiation [37].

Lim et al. reported *CLU* mRNA expression in ameloblastomas; however, CLU protein expression has been confirmed in only a limited number of cases [38]. However, the correlation between the expression of CLU and those of other proteins remains uninvestigated. Our findings demonstrate that all SYP-positive samples were positive for CLU. In contrast, most CD56-positive specimens were CLU-negative. Statistically significant correlations were only observed between CLU and SYP expression. Considering the known functions of CLU, our results suggest that it is closely linked to the differentiation of a subset of ameloblastoma cells into neuroendocrine-like cells that express SYP and INSM1.

One limitation of this study is the small number of specimens with SYP- and INSM1-positive cells, as well as the limited availability of samples for detailed analyses, including TEM and mRNA studies, due to prolonged fixation or small size of specimens. This limitation restricts our ability to draw comprehensive conclusions regarding the extent and nature of neuroendocrine differentiation in ameloblastomas. To gain a deeper insight into the underlying molecular mechanisms, collecting a larger number of appropriate specimens that consistently express SYP and INSM1 is necessary. Additionally, incorporating advanced techniques, such as molecular profiling, could help further elucidate the biological pathways involved and refine our understanding of these unique cellular features in ameloblastomas.

## 5. Conclusions

This study is the first to suggest neuroendocrine differentiation in a small subset of ameloblastomas, characterized by SYP positivity and CgA negativity, along with INSM1 immunoexpression, in line with the recent understanding of neuroendocrine characteristics in the WHO classification. CD56 demonstrated a limited impact, as most CD56-positive cases lacked neuroendocrine differentiation. However, SYP-positive cases were exclusively positive for both CD56 and CLU, indicating that CLU might play a supportive role in neuroendocrine differentiation. Despite these findings, the molecular mechanisms remain unclear. Further research using more extensive specimen analysis is needed to better understand the unique neuroendocrine characteristics of ameloblastomas.

## Figures and Tables

**Table 1:** Panel of primary antibodies.

Antibody	Source	Clone	Code	Retrieval	Dilution
CD56	Agilent	123C3	M7304	HIER, pH6.0	1:100
SYP	Agilent	M0776	M7315	HIER, pH6.0	1:20
CgA	Leica	PA0515	PA0515	HIER, pH6.0	1:200
INSM1	Santa Cruz	A-8	sc-271408	HIER, pH9.0	1:100
CLU	Biocare	41D	CM218A	HIER, pH6.0	1:100

SYP: synaptophysin; CgA: chromogranin A; INSM1; insulinoma-associated protein 1; CLU: clusterin; HIER: heat-induced epitope retrieval; Agilent: Agilent Technologies, Santa Clara, CA, USA; Leica: Leica Biosystems, Nussloch, Germany; Santa Cruz: Santa Cruz Biotechnology, Inc., Dallas, TX, USA; Biocare: Biocare Medical, Pacheco, CA, USA.

**Table 2:** Primer sets.

Gene Name	Sequence	Size (bp)
*NCAM1*	F: 5′-GTGTGGTTACAGGCGAGGAT-3′	191
	R: 5′-GATGACATCTCGGCCTTTGT-3′	
*SYP*	F: 5′-GGGCGTGACTTCAGACTCTC-3′	181
	R: 5′-CAAGACCACCTTGGGTCCTA-3′	
*CHGA*	F: 5′-CTACGCGCCTTGTCTCCTAC-3′	153
	R: 5′-AGTTGTGCCCAGTGGATAGG-3′	
*GAPDH*	Not disclosed (Commercial primer set; Takara Bio Inc., Primer set ID: HA067812)	138

**Table 3:** Summary of ameloblastomas with immunoexpression of CD56 and SYP.

Case	Type	Sex	Age	CD56	SYP	CgA	INSM1	CLU
4	Conventional	M	33	+	+	−	−	+
25	Unicystic	M	57	+	+	−	+	+
30	Conventional	M	81	+	+	−	+	+
32	Unicystic	F	44	+	+	−	+	+
36	Conventional	M	70	+	+	−	+	+

SYP: synaptophysin; CgA: chromogranin A; INSM1: insulinoma-associated protein; CLU: clusterin.

**Table 4:** Relationships between histological subtypes and neuroendocrine maker expression.

	Conventional	Unicystic	*p*-Value
CD56(−)	8	2	0.142
CD56 (+)	13	13	
SYP (−)	18	13	1
SYP (+)	3	2	
CLU (−)	18	10	0.236
CLU (+)	3	5	

CLU: clusterin; SYP: synaptophysin.

**Table 5:** Relationships between clusterin and synaptophysin expression.

	CLU (−)	CLU (+)	*p*-Value
CD56 (−)	10	0	0.0756
CD56 (+)	18	8	
SYP (−)	28	3	<0.001
SYP (+)	0	5	

CLU: clusterin; SYP: synaptophysin.

## Data Availability

All data were included in this manuscript and Supplemental Table S1.

## Supplementary Materials

The following supporting information can be downloaded at https://www.mdpi.com/article/10.3390/cells14030224/s1, Supplemental Table S1: Summary of clinical, histological, and immunohistochemical results.

## References

[1] WHO Classification of Tumours Editorial Board (2023). Head and Neck Tumors.

[2] Lanier L.L., Testi R., Bindl J., Phillips J.H. (1989). Identity of Leu-19 (CD56) Leukocyte Differentiation Antigen and Neural Cell Adhesion Molecule. J. Exp. Med..

[3] Uezumi A., Fukada S., Yamamoto N., Ikemoto-Uezumi M., Nakatani M., Morita M., Yamaguchi A., Yamada H., Nishino I., Hamada Y. (2014). Identification and Characterization of PDGFRα+ Mesenchymal Progenitors in Human Skeletal Muscle. Cell Death Dis..

[4] Huang R., Yu L., Zheng C., Liang Q., Suye S., Yang X., Yin H., Ren Z., Shi L., Zhang Z. (2020). Diagnostic Value of Four Neuroendocrine Markers in Small Cell Neuroendocrine Carcinomas of the Cervix: A Meta-Analysis. Sci. Rep..

[5] Pyo J.-S., Kim D.-H., Yang J. (2018). Diagnostic Value of CD56 Immunohistochemistry in Thyroid Lesions. Int. J. Biol. Markers.

[6] Kusafuka K., Hirobe K., Wato M., Tanaka A., Nakajima T. (2011). CD56 Expression Is Associated with Neuroectodermal Differentiation in Ameloblastomas: An Immunohistochemical Evaluation in Comparison with Odontogenic Cystic Lesions. Med. Mol. Morphol..

[7] Cairns L., Naidu A., Robinson C.M., Sloan P., Wright J.M., Hunter K.D. (2010). CD56 (NCAM) Expression in Ameloblastomas and Other Odontogenic Lesions. Histopathology.

[8] Jaafari-Ashkavandi Z., Dehghani-Nazhvani A., Razmjouyi F. (2014). CD56 Expression in Odontogenic Cysts and Tumors. J. Dent. Res. Dent. Clin. Dent. Prospect..

[9] Megyesfalvi Z., Barany N., Lantos A., Valko Z., Pipek O., Lang C., Schwendenwein A., Oberndorfer F., Paku S., Ferencz B. (2022). Expression Patterns and Prognostic Relevance of Subtype-Specific Transcription Factors in Surgically Resected Small-Cell Lung Cancer: An International Multicenter Study. J. Pathol..

[10] Baine M.K., Hsieh M.-S., Lai W.V., Egger J.V., Jungbluth A.A., Daneshbod Y., Beras A., Spencer R., Lopardo J., Bodd F. (2020). SCLC Subtypes Defined by ASCL1, NEUROD1, POU2F3, and YAP1: A Comprehensive Immunohistochemical and Histopathologic Characterization. J. Thorac. Oncol..

[11] Perez-Ordoñez B. (2018). Neuroendocrine Carcinomas of the Larynx and Head and Neck: Challenges in Classification and Grading. Head. Neck Pathol..

[12] Mete O., Wenig B.M. (2022). Update from the 5th Edition of the World Health Organization Classification of Head and Neck Tumors: Overview of the 2022 WHO Classification of Head and Neck Neuroendocrine Neoplasms. Head Neck Pathol.

[13] La Rosa S., Sessa F., Uccella S. (2016). Mixed Neuroendocrine-Non-neuroendocrine Neoplasms (MiNENs): Unifying the Concept of a Heterogeneous Group of Neoplasms. Endocr. Pathol..

[14] Kanda Y. (2013). Investigation of the Freely Available Easy-to-Use Software “EZR” for Medical Statistics. Bone Marrow Transpl..

[15] Juhlin C.C. (2021). Second-Generation Neuroendocrine Immunohistochemical Markers: Reflections from Clinical Implementation. Biology.

[16] Yirmibeş S., Adım Ş.B., Saraydaroğlu Ö. (2023). CD56 and Smooth Muscle Actin Immunoreactivity in Basal Cell Carcinomas: Are They Indicators of Differentiation or Do They Hold a Diagnostic Use?. J. Cutan. Pathol..

[17] Collins A.P., Mubarak N., Hemaidan H.S., Hemaidan S.M., Hemaidan A. (2021). Malignant Ameloblastoma with Hepatic Metastasis in a 38-Year-Old Haitian Woman. Am. J. Case Rep..

[18] López-Otín C., Blasco M.A., Partridge L., Serrano M., Kroemer G. (2023). Hallmarks of Aging: An Expanding Universe. Cell.

[19] Mohan K., Gasparoni G., Salhab A., Orlich M.M., Geffers R., Hoffmann S., Adams R.H., Walter J., Nordheim A. (2023). Age-Associated Changes in Endothelial Transcriptome and Epigenetic Landscapes Correlate With Elevated Risk of Cerebral Microbleeds. J. Am. Heart Assoc..

[20] Matthiessen M.E., Vedtofte P., Rømert P. (1980). Morphology of a Simple Ameloblastoma Related to the Human Enamel Organ. Scand. J. Dent. Res..

[21] Tsutsumi Y. (2018). Electron Microscopic Study Using Formalin-Fixed, Paraffin-Embedded Material, with Special Reference to Observation of Microbial Organisms and Endocrine Granules. Acta Histochem. Cytochem..

[22] Capella C., Usellini L., Papotti M., Macrì L., Finzi G., Eusebi V., Bussolati G. (1990). Ultrastructural Features of Neuroendocrine Differentiated Carcinomas of the Breast. Ultrastruct. Pathol..

[23] Panneer Selvam S., Ponniah I. (2018). Expression of Ameloblastin in the Human Tooth Germ and Ameloblastoma. Oral. Dis..

[24] Crivelini M.M., Felipini R.C., Miyahara G.I., de Sousa S.C.O.M. (2012). Expression of Odontogenic Ameloblast-Associated Protein, Amelotin, Ameloblastin, and Amelogenin in Odontogenic Tumors: Immunohistochemical Analysis and Pathogenetic Considerations. J. Oral. Pathol. Med..

[25] Tsujigiwa H., Nagatsuka H., Han P.P., Gunduz M., Siar C.H., Oida S., Nagai N. (2005). Analysis of Amelogenin Gene (AMGX, AMGY) Expression in Ameloblastoma. Oral. Oncol..

[26] Rindi G., Mete O., Uccella S., Basturk O., La Rosa S., Brosens L.A.A., Ezzat S., de Herder W.W., Klimstra D.S., Papotti M. (2022). Overview of the 2022 WHO Classification of Neuroendocrine Neoplasms. Endocr Pathol.

[27] Rooper L.M., Bishop J.A., Westra W.H. (2018). INSM1 Is a Sensitive and Specific Marker of Neuroendocrine Differentiation in Head and Neck Tumors. Am. J. Surg. Pathol..

[28] Mukhopadhyay S., Dermawan J.K., Lanigan C.P., Farver C.F. (2019). Insulinoma-Associated Protein 1 (INSM1) Is a Sensitive and Highly Specific Marker of Neuroendocrine Differentiation in Primary Lung Neoplasms: An Immunohistochemical Study of 345 Cases, Including 292 Whole-Tissue Sections. Mod. Pathol..

[29] Wyatt A.R., Yerbury J.J., Wilson M.R. (2009). Structural Characterization of Clusterin-Chaperone Client Protein Complexes. J. Biol. Chem..

[30] Wyatt A.R., Yerbury J.J., Berghofer P., Greguric I., Katsifis A., Dobson C.M., Wilson M.R. (2011). Clusterin Facilitates in Vivo Clearance of Extracellular Misfolded Proteins. Cell. Mol. Life Sci..

[31] Rodríguez-Rivera C., Garcia M.M., Molina-Álvarez M., González-Martín C., Goicoechea C. (2021). Clusterin: Always Protecting. Synthesis, Function and Potential Issues. Biomed. Pharmacother..

[32] Andersen C.L., Schepeler T., Thorsen K., Birkenkamp-Demtröder K., Mansilla F., Aaltonen L.A., Laurberg S., Ørntoft T.F. (2007). Clusterin Expression in Normal Mucosa and Colorectal Cancer. Mol. Cell. Proteom..

[33] Czeczok T.W., Stashek K.M., Maxwell J.E., O’Dorisio T.M., Howe J.R., Hornick J.L., Bellizzi A.M. (2018). Clusterin in Neuroendocrine Epithelial Neoplasms: Absence of Expression in a Well-Differentiated Tumor Suggests a Jejunoileal Origin. Appl. Immunohistochem. Mol. Morphol..

[34] Xue T., Wei L., Zha D.-J., Qiao L., Lu L.-J., Chen F.-Q., Qiu J.-H. (2015). Exposure to Acoustic Stimuli Promotes the Development and Differentiation of Neural Stem Cells from the Cochlear Nuclei through the Clusterin Pathway. Int. J. Mol. Med..

[35] Landin M.A.D.S.S., Shabestari M., Babaie E., Reseland J.E., Osmundsen H. (2012). Gene Expression Profiling during Murine Tooth Development. Front. Genet..

[36] Osmundsen H., Landin M.A., From S.H., Kolltveit K.M., Risnes S. (2007). Changes in Gene-Expression during Development of the Murine Molar Tooth Germ. Arch. Oral. Biol..

[37] Khan Q.-E.-S., Sehic A., Khuu C., Risnes S., Osmundsen H. (2013). Expression of Clu and Tgfb1 during Murine Tooth Development: Effects of in-Vivo Transfection with Anti-miR-214. Eur. J. Oral. Sci..

[38] Lim J., Ahn H., Min S., Hong S.-D., Lee J.-I., Hong S.-P. (2006). Oligonucleotide Microarray Analysis of Ameloblastoma Compared with Dentigerous Cyst. J. Oral. Pathol. Med..

